# Prevalence of Sarcopenic Obesity and Factors Influencing Body Composition in Persons with Spinal Cord Injury in Japan

**DOI:** 10.3390/nu15020473

**Published:** 2023-01-16

**Authors:** Ryu Ishimoto, Hirotaka Mutsuzaki, Yukiyo Shimizu, Hiroshi Kishimoto, Ryoko Takeuchi, Yasushi Hada

**Affiliations:** 1 Doctoral Program in Medical Sciences, Graduate School of Comprehensive Human Sciences, University of Tsukuba, Tsukuba 305-8575, Japan; 2 Department of Physical Medicine and Rehabilitation, Ibaraki Prefectural University of Health Sciences Hospital, Ami 300-0331, Japan; 3 Department of Orthopedic Surgery, Ibaraki Prefectural University of Health Sciences Hospital, Ami 300-0331, Japan; 4 Center for Medical Science, Ibaraki Prefectural University of Health Sciences, Ami 300-0394, Japan; 5 Department of Rehabilitation Medicine, Faculty of Medicine, University of Tsukuba, Tsukuba 305-8575, Japan

**Keywords:** spinal cord injury, sarcopenic obesity, body composition, dual-energy X-ray absorptiometry, obesity, sarcopenia

## Abstract

This study aims to investigate the prevalence of sarcopenic obesity and factors influencing body composition in persons with spinal cord injury (SCI) in Japan. Adults with SCI aged ≥ 20 years who underwent whole-body dual-energy X-ray absorptiometry between 2016 and 2022 were retrospectively analyzed. Data from 97 patients were examined. The primary outcome was appendicular skeletal muscle mass (ASM). Multiple linear regression analysis was conducted to assess factors influencing the lean and adipose indices in persons with SCI. Sarcopenia, obesity, and sarcopenic obesity were prevalent in 76%, 85%, and 64% of patients, respectively. Multivariate linear regression analysis revealed that sex (β = 0.34, *p* < 0.001), lesion level (β = 0.25, *p* = 0.007), severity (β = 0.20, *p* = 0.043), and ability to walk (β = 0.29, *p* = 0.006) were independently associated with ASM. Sex (β = −0.63, *p* < 0.001) was independently associated with percent body fat. In conclusion, sarcopenia, obesity, and sarcopenic obesity were prevalent among patients with SCI in Japan. Female sex, tetraplegia, motor-complete injury, and inability to walk were risk factors for sarcopenia, whereas female sex was a risk factor for obesity in persons with SCI. A routine monitoring of body composition is necessary, especially among those with multiple risk factors, to identify individuals in need of preventive and therapeutic interventions.

## 1. Introduction

Obesity is a complication commonly observed in persons with spinal cord injury (SCI) and is more prevalent in individuals with SCI than in the general population [1,2]. An increase in fat accumulation, particularly in the abdominal region, is associated with multiple health problems, including cardiovascular diseases and metabolic syndrome [3,4]. Additionally, obesity in those with traumatic SCI is associated with a reduction in function, mobility, and quality of life [4].

A loss of muscle mass and function can synergistically worsen the adverse effects of obesity. Sarcopenia refers to the age-related decline in lean-soft-tissue mass and muscle strength or physical function [5,6]. Sarcopenic obesity (i.e., the presence of both sarcopenia and obesity) is a strong predictor of all-cause mortality and activities of daily living (ADL) performance in the elderly [7,8,9]. Its negative impacts on health include an increased risk of frailty and falls, functional decline, physical dependency, institutionalization, and mortality [6,10]. A decline in lean-soft-tissue mass similarly occurs in patients with SCI as a result of SCI-related muscle atrophy, deconditioning, and physical inactivity [11,12,13]. Hence, complications resulting from sarcopenia and sarcopenic obesity in SCI are thought to be similar to those reported in the geriatric population, with accelerated time courses.

However, data on sarcopenic obesity in the SCI population are scarce. The definition and diagnostic criteria of sarcopenic obesity remain unclear [14]. In addition, the assessment of sarcopenia and sarcopenic obesity in individuals with SCI requires special considerations because disabilities resulting from SCI make accurate measurement of muscle function (strength and performance) challenging [11]. Without evaluating muscle function, Pelletier et al. [7] investigated the lean and fat tissue mass of individuals with chronic SCI by using whole-body dual-energy X-ray absorptiometry (DXA). They reported that the prevalence of sarcopenia and sarcopenic obesity were 56.6% and 41.9%, respectively.

The Japanese population is aging rapidly, and the causes and incidence rates of traumatic SCI have been changing. Worldwide, the leading causes of injury are road traffic accidents followed by falls, and the average age of patients is 40 years [15]. On the other hand, a recent nationwide Japanese survey revealed that falling on a level surface was the leading cause of injury [16]; the incidence of traumatic SCI peaked in the seventh decade. In addition, the rate of cervical and incomplete injuries was increasing with the aging population. Hence, the frequency and magnitude of sarcopenia and sarcopenic obesity in the Japanese population with SCI may be different from those in individuals with SCI from other countries. However, most studies regarding the body composition of individuals with SCI were conducted in Western countries [4], and only a few have been conducted in Japan with limited sample sizes [17,18,19,20].

Therefore, the present study aims to evaluate the lean and adipose indices of Japanese persons with SCI by DXA-measured outcomes. The prevalence and magnitude of sarcopenia, obesity, and sarcopenic obesity were examined (without assessing muscle function). In addition, factors associated with these indices were investigated.

## 2. Materials and Methods

### 2.1. Participants and Setting

Patients were referred to our rehabilitation hospital for post-acute care and were treated in inpatient and outpatient settings. This cross-sectional study was conducted in our post-acute rehabilitation hospital with 120 beds and enrolled patients with SCI who underwent whole-body DXA between September 2016 and August 2022. Patients aged ≥ 20 years who had motor dysfunction with either tetraplegia or paraplegia were included. Those with missing data were excluded from the analysis.

### 2.2. Data Collection

Basic data, including sex, age, diagnosis, and duration (in years) of injury, were obtained through medical chart abstraction. Lesion level (tetraplegia/paraplegia) and severity of injury (motor-complete/motor-incomplete) were determined using the American Spinal Injury Association Impairment Scale (AIS) criteria based on the International Standards for Neurological Classification of SCI [21]. Tetraplegia was defined as lesion level C2 to Th1, and paraplegia was defined as level Th2 or below. AIS A or B was categorized as “motor-complete,” whereas AIS C or D was categorized as “motor-incomplete.”

The AIS classifies complete and incomplete SCI [21]. A complete SCI was defined as the absence of all motor and sensory function, including sacral segments S4–5 (classified as AIS A). For incomplete SCI, some degree of motor or sensory function was preserved below the injury site. Patients with some sensory function but no motor function preserved were classified as AIS B, while those with a motor grade of less than 3 below the neurologic level of injury were classified as AIS C. In addition, patients with a motor grade of at least 3 below the neurologic level of injury were classified as AIS D.

The presence of dysphagia was identified via medical chart review. If the patients were tube-fed or provided with a texture-modified meal to support their swallowing function, they were considered to have difficulty swallowing; otherwise, the patients were deemed to have no problem with swallowing.

The ability to walk was determined by reviewing rehabilitation records. If the patients could walk at any time of the day with or without orthoses or assistive devices, they were counted as walkers. If the patients could not walk using any of these kinds of devices, they were counted as non-walkers.

### 2.3. Outcome Measurements

The primary outcome was appendicular skeletal muscle mass (ASM), and the secondary outcome was percent body fat (%BF). These were measured using whole-body DXA with Horizon A models (Hologic Inc., Marlborough, MA, USA). Commercially available APEX software version 5.6.04 (Hologic Inc.) was used to obtain and analyze the whole-body scans.

A nurse measured the patients’ height and weight using a tape measure and electronic scale, respectively, in the supine position in both the inpatient and outpatient settings. These data were obtained through medical chart abstraction. Body mass index (BMI) was calculated as body mass divided by the square of the height value (kg/m^2^).

### 2.4. Criteria for Defining Sarcopenia, Obesity, and Sarcopenic Obesity

The criteria for the general population were applied to determine the prevalence of sarcopenia. Sarcopenia is diagnosed when low ASM and low hand grip strength are present [5]; however, hand grip strength is difficult to accurately measure in tetraplegic patients. Hence, based on a previous study [7], only ASM was used as the criterion for identifying patients with sarcopenia in this study. The cutoff values were ASM < 7.0 kg/m^2^ for men and <5.4 kg/m^2^ for women, as recommended by the Asian Working Group for Sarcopenia 2019 consensus [5].

As for obesity, multiple criteria were introduced. The American Association of Clinical Endocrinology recommends the use of %BF for diagnosing obesity [6,22]. With this criterion, obesity is defined as %BF > 25% in men and > 35% in women. Nash et al. recommend %BF > 22% in men and > 35% in women as the SCI-specific cutoff values for obesity identification [23]. The criteria for obesity in the general Japanese population are as follows: BMI ≥ 25 kg/m^2^; visceral adipose tissue (VAT) ≥ 100 cm^2^; and waist circumference (WC) ≥ 85 cm in men and ≥ 90 cm in women [24]. In our study, obesity was diagnosed using several criteria: (1) %BF > 22% for men, > 35% for women; (2) %BF > 25% for men, > 35% for women; (3) BMI ≥ 25 kg/m^2^; and (4) VAT ≥ 100 cm^2^.

There exists no consensus regarding the diagnostic criteria for sarcopenic obesity in the general population. Based on a previous study [7], sarcopenic obesity was identified if the criteria for obesity and sarcopenia were met.

### 2.5. Sample Size Calculation

The optimal number of samples was calculated using G*Power 3.1. The effective size for ASM was calculated as 0.89 using data from a previous study [7]. Based on these data, the sample size in the *t*-test was calculated as 42 when the α error was 0.05 and (1-β) error probability was 0.8.

### 2.6. Statistical Analysis

Data are expressed as mean (standard deviation) for parametric data, as median with 25–75% (interquartile range) for nonparametric data, and as numerical values (%) for categorical data. The participants were divided into groups according to lesion level (tetraplegia/paraplegia), severity (motor-complete/motor-incomplete), sex (female/male), and walking ability (walkers/non-walkers). The Shapiro–Wilk test was used to assess normality. Levene’s test was performed to assess the equality of variance. Depending on the variables, the *t*-test, Welch’s *t*-test, Mann–Whitney *U* test, and chi-squared test were performed to compare the demographic and impairment characteristics. Multiple linear regression analysis was conducted to determine the association of patients’ characteristics (sex, age, duration of injury, lesion level, severity, ability to walk, presence of dysphagia, and etiology of injury) with DXA-measured sarcopenia and obesity-related outcomes (ASM, %BF, whole-body lean mass, whole-body fat mass). The variance inflation factor (VIF) was used to assess multicollinearity. VIF values ranging from 1 to 10 were indicative of the absence of multicollinearity. IBM SPSS Statistics version 28.0 (IBM, Tokyo, Japan) was used to analyze the data, with statistical significance set at *p*-values < 0.05.

### 2.7. Ethical Considerations

The present study was conducted in accordance with the principles embodied in the Declaration of Helsinki and the Ethical Guidelines for Medical and Health Research Involving Human Subjects. The Ethics Committee of the Ibaraki Prefectural University of Health Sciences approved this study (approval no: e341; date of approval: 29 March 2022). Owing to the retrospective nature of this study, an opt-out procedure for recruitment was instituted by announcing the research project in the hospital and via its webpage, and the need for the acquisition of written informed consent from patients was waived. The participants could withdraw from the study at any time through the opt-out procedure.

## 3. Results

### 3.1. Participants

A total of 112 patients with SCI underwent whole-body DXA during the study period. Of these, four patients aged < 20 years and eight patients with missing data were excluded. Additionally, those with severe dysfunction other than paralysis (cancer, *n* = 1; cerebral palsy, *n* = 1; and Parkinson’s disease, *n* = 1) were excluded from the analysis. The remaining 97 patients were included in this study.

The etiology of SCI is summarized in Table 1. Among the patients, 79% and 21% had traumatic and non-traumatic SCI, respectively. Non-traumatic SCI included spinal cord infarction (*n* = 11), spinal epidural hematoma (*n* = 4), spinal epidural abscess (*n* = 1), and transverse myelitis (*n* = 4).

Table 2 presents the patients’ characteristics. According to lesion level, the groups were similar with respect to all variables, except for the etiology of injury (*p* < 0.001), weight (*p* = 0.002), and BMI (*p* < 0.001). Non-traumatic SCI was more prevalent in patients with paraplegia (45%) than in those with tetraplegia (5%). Patients with paraplegia had a greater body weight and BMI (66.8 [58.9–74.7] kg; 24.4 [22.4–26.4] kg/m^2^, respectively) than those with tetraplegia (59.0 [51.9–66.1] kg; 21.8 [19.4–24.2] kg/m^2^, *p* = 0.002; *p* < 0.001, respectively). According to severity, the age at the onset of injury was higher in patients with motor-incomplete than in those with motor-complete SCI (62.5 [52.4–72.6] vs. 52.0 [42.0–62.0], *p* = 0.005). The duration of injury was longer in patients with motor-complete than in those with motor-incomplete SCI (4.4 [0–11.9] vs. 0.3 [0–3.0], *p* < 0.001). The proportion of walkers was significantly higher in patients with motor-incomplete than in those with motor-complete SCI (71% vs. 10%, *p* < 0.001). According to sex, the groups were similar with respect to all variables, except for height (*p* < 0.001) and weight (*p* = 0.011). Males were taller and heavier (168.2 ± 6.2 cm, 63.5 [55.6–71.4] kg, respectively) than females (154.5 ± 6.3 cm, 55.5 [47.5–63.5] kg, respectively). According to walking ability, the duration of injury was longer in non-walkers than in walkers (3.9 [0–9.7] vs. 0.3 [0–1.5] years, *p* = 0.002). The proportion of motor-complete injury was significantly higher in non-walkers than in walkers (60% vs. 6%, *p* < 0.001). Body weight was greater in walkers than in non-walkers (65.3 [56.7–73.8] kg vs. 59.0 [52.0–66.1] kg, *p* = 0.044).

### 3.2. Lean Tissue and Fat Mass

DXA-measured body composition outcomes are shown in Table 3. Regarding lean tissue mass indices, lean tissue mass in the whole body, trunk, and arms were significantly lower in patients with tetraplegia than in those with paraplegia (*p* = 0.002; *p* < 0.001; *p* < 0.001). ASM was significantly lower in patients with paraplegia than in those with tetraplegia (*p* = 0.049). According to severity, lean tissue mass in the whole body and legs, % whole-body lean mass, and ASM were significantly lower in patients with motor-complete than in those with motor-incomplete SCI (*p* = 0.012; *p* < 0.001; *p* = 0.010; *p* < 0.001, respectively). According to sex, the lean tissue mass in the whole body, trunk, arms, and legs, % whole-body lean mass, and ASM were significantly lower in females than in males (all *p* < 0.001). According to walking ability, the lean tissue mass in the whole body, trunk, and legs, % whole-body lean mass, and ASM were significantly lower in non-walkers than in walkers (*p* < 0.001; *p* = 0.016; *p* < 0.001; *p* = 0.003; *p* < 0.001, respectively).

Regarding adiposity indices, patients with paraplegia had greater whole-body fat mass than those with tetraplegia (*p* = 0.020). Regional fat mass distributions differed between the groups. Trunk fat mass and VAT were significantly higher in patients with paraplegia than in those with tetraplegia (*p* = 0.023; *p* = 0.034, respectively). In contrast, the fat mass of the arms and legs showed no significant difference. Except for %BF, no significant differences in adipose indices were detected between the patients with motor-complete and motor-incomplete SCI; those with motor-complete exhibited significantly greater %BF than those with motor-incomplete SCI (*p* = 0.012). According to sex, females had significantly higher whole-body fat mass and %BF than males (*p* = 0.003; *p* < 0.001, respectively). Regional fat mass distributions were also different across all regions; the fat mass of the trunk, arms, and legs were significantly higher in females than in males (*p* = 0.005; *p* = 0.004; *p* < 0.001, respectively). No significant difference in VAT was observed between males and females. According to walking ability, %BF was significantly higher in non-walkers than in walkers (*p* = 0.005). No significant differences were observed in other adipose indices.

### 3.3. Prevalence of Sarcopenia and Obesity

Table 4 presents the prevalence of sarcopenia, obesity, and sarcopenic obesity based on the general and SCI-specific criteria. Of the patients, 76% were identified as having sarcopenia (as defined by the ASM threshold). The prevalence of obesity and sarcopenic obesity varied depending on the criteria. The prevalence of obesity was 68% (as defined by %BF > 25% in males and >35% in females), 85% (as defined by %BF > 22% in males and >35% in females), 29% (as defined by BMI ≥ 25 kg/m^2^), and 64% (as defined by VAT ≥ 100 cm^2^). The prevalence of sarcopenic obesity was 51% (as defined by %BF > 25% in males and > 35% in females), 64% (as defined by %BF > 22% in males and > 35% in females), 11% (as defined by BMI ≥ 25 kg/m^2^), and 44% (as defined by VAT ≥ 100 cm^2^).

### 3.4. Factors Influencing Lean Tissue and Fat Mass

Table 5 shows the results of multivariate linear regression analysis. Multicollinearity was not observed among the variables. The results indicate that female sex (β = 0.34, *p* < 0.001), tetraplegia (β = 0.25, *p* = 0.007), motor-complete injury (β = 0.20, *p* = 0.043), and inability to walk (β = 0.29, *p* = 0.006) were independently associated with lower ASM. Female sex (β = −0.63, *p* < 0.001) was independently associated with higher %BF. Female sex (β = 0.50, *p* < 0.001), older age (β = −0.22, *p* = 0.005), tetraplegia (β = 0.31, *p* < 0.001), and complete injury (β = 0.20, *p* = 0.031) were independently associated with lower whole-body lean mass. Female sex (β = −0.28, *p* = 0.006) and paraplegia (β = 0.28, *p* = 0.018) were independently associated with higher whole-body fat mass.

## 4. Discussion

This study examined the prevalence of sarcopenia, obesity, and sarcopenic obesity in patients with SCI in Japan and investigated the factors influencing these outcomes. Our results revealed two clinical observations: (1) sarcopenia, obesity, and sarcopenic obesity were prevalent among individuals with SCI; and (2) female sex, tetraplegia, motor-complete injury, and inability to walk were identified as risk factors for muscle atrophy comparable to sarcopenia in persons with SCI. Furthermore, female sex was identified as a risk factor for obesity in persons with SCI.

Sarcopenia has a reported prevalence of 1–29% in the community-dwelling elderly, 14–33% in long-term care facility populations, and 10% in acute care hospitals [25]. Studies from Japan have reported that patients undergoing rehabilitation in convalescent rehabilitation wards account for approximately 50% [26]. In the general Japanese population, the prevalence of obesity (defined by BMI ≥ 25 kg/m^2^) is 33.0% in males and 23.3% in females [27]. A previous study conducted on 376 patients with stroke in a rehabilitation hospital in Japan showed a prevalence of 32%, 17%, and 28% for sarcopenia, obesity, and sarcopenic obesity, respectively [28].

Compared with these data, the prevalence of sarcopenia, obesity, and sarcopenic obesity in our patients with SCI was high. Based on the thresholds designed for the general population, 76% of our patients were identified as having sarcopenia. The prevalence of obesity and sarcopenic obesity varied depending on the criteria for obesity, but up to 85% of our patients were identified as having obesity (when the SCI-specific threshold for obesity was applied). In addition, up to 64% of our patients satisfied both the sarcopenia and obesity thresholds, which were thought to be equivalent to those of sarcopenic obesity. Using different guidelines and criteria, Pelletier et al. yielded similar results in patients with chronic SCI and reported a prevalence of 72%, 57%, and 42% for obesity, sarcopenia, and sarcopenic obesity, respectively [7].

Obesity is a major risk factor for cardiometabolic diseases in the general and SCI populations [29,30,31,32]. In particular, excessive abdominal obesity has been reported to be associated with cardiovascular diseases, insulin resistance, and dyslipidemia [30,33]. Sarcopenia synergistically worsens the adverse effects of obesity, leading to unfavorable health conditions, such as an increased risk of frailty and falls, functional decline, physical dependency, institutionalization, and mortality [6,10]. Hence, the identification of individuals with risk factors associated with sarcopenia, obesity, and sarcopenic obesity among patients with SCI is crucial to preventing these adverse changes.

Multiple factors are responsible for changes in body composition following SCI. For example, sex is an essential determinant of body composition. A previous study investigating sex differences in body composition in the able-bodied population revealed that females had higher total body fat and percent fat mass but lower lean mass than males [34]. Changes in body composition at menopause in females must also be considered when an increase in body weight and fat mass but a decrease in fat-free mass pronouncedly occur [6,35]. Data on the population with SCI are scarce, as SCI rarely occurs in females. In our study, female sex was independently associated with lower lean mass indices (i.e., ASM and whole-body lean mass) and higher adipose indices (i.e., %BF and whole-body fat mass). These results are consistent with the finding observed in the able-bodied population: females have a higher risk of sarcopenia and obesity than males.

Aging and duration of injury can also influence body composition. A previous study showed that advancing age and injury duration were correlated with less %lean mass and more adiposity in the population with SCI [13]. A Korean study also reported a positive association with duration and %BF [36]. In our study, age was negatively associated with whole-body lean mass (β = −0.22); therefore, advancing age was considered a risk factor for the reduction in whole-body lean mass. However, in our study, duration was not independently associated with lean and adipose indices. Because age and duration are variables associated with each other, these two are “difficult to detangle” [4]. Further studies are thus required to determine the effects of aging and the duration of injury on body composition in the population with SCI.

Lesion level (as a determinant of either tetraplegia or paraplegia) and severity are also associated with body composition. Individuals with tetraplegia have been reported to have lower lean mass than those with paraplegia [4,7,13]. Additionally, individuals with complete injury have lower lean [7] and higher fat composition than those with incomplete injury [13,36]. Congruent with previous reports, we found that lesion level was independently associated with ASM, and whole-body lean mass. Therefore, individuals with tetraplegia were more at risk of sarcopenia, and lower whole-body lean mass than those with paraplegia. Furthermore, severity was independently associated with ASM and whole-body lean mass. Hence, individuals with motor-complete injuries have a higher risk of sarcopenia and lower whole-body lean mass than those with motor-incomplete injuries.

In contrast, previous studies reported that individuals with tetraplegia had higher fat tissue mass than those with paraplegia [4,7,13]. However, our study found significantly greater fat tissue mass in patients with paraplegia than in those with tetraplegia. In addition, paraplegia was independently associated with higher whole-body fat mass. This discrepancy may be attributable to differences in our patients’ characteristics. Our study included patients in the subacute to chronic phase; thus, body composition might have still been changing [37]. Additionally, our patients were much older (median age: 64 years) and more patients suffered from motor-incomplete injuries than those included in previous studies. Racial, ethnic, and lifestyle-related differences might also have influenced our results, as all of our patients were Japanese. However, a possible reason for our results could be differences in caloric intake relative to energy needs. Following SCI, the basal metabolic rate decreases as the muscles atrophy below the lesion level [38]. Because the loss of metabolically active tissues is more significant in individuals with tetraplegia than in those with paraplegia, the energy requirement is lower in individuals with tetraplegia than in those with paraplegia. On the other hand, persons with paraplegia have more independence to freely eat than those with tetraplegia, who are more likely to depend on caregivers for ADLs [2]. Hence, more patients with paraplegia might have been exposed to excessive caloric intake relative to their energy needs, resulting in fat tissue mass gain.

Irrespective of differences in fat tissue mass between the groups, the prevalence of obesity was high in both groups. Thus, preventive and therapeutic interventions are necessary. With caloric restriction to match energy needs, exercise interventions for increasing energy expenditure are the hallmark of the prevention and treatment of sarcopenic obesity. Evidence supports the overall benefit of exercise interventions in the general population [6]. Despite lacking evidence for the population with SCI, some studies reported promising results. For instance, a previous study reported that supervised physical activity in individuals with SCI resulted in favorable changes in body composition [39]. Another study investigated the effect of 6-week locomotor training using a robotic exoskeleton and reported an increase in lean tissue mass and a decrease in fat tissue mass in individuals with chronic SCI [40]. In 2021, Asselin et al. evaluated the effect of walking exercise using a powered exoskeleton and reported a reduction in total body fat mass in individuals with chronic SCI [41]. Our study revealed that walking ability was independently associated with ASM. Hence, wheelchair-dependent individuals were thought to be more at risk of sarcopenia. This also suggested that walking exercises with or without any assistive device might effectively slow down or prevent the progression of the undesired loss of appendicular lean mass. Nonetheless, the walking ability in our study might have reflected other confounding variables, such as physical activity level, and more studies are therefore required to determine the effects of walking ability on body composition. Furthermore, routine monitoring of body composition was considered necessary, especially in those with factors such as tetraplegia, motor-complete injury, and inability to walk, to identify individuals in need of preventive and therapeutic interventions.

Finally, it is worthwhile to mention that BMI is not a useful measure of adiposity in individuals with SCI. According to the World Health Organization, the threshold for obesity in the general population is a BMI of > 30 kg/m^2^. Based on this definition, obesity is prevalent in only a small percentage of the Japanese population [24,42]. Therefore, the threshold for obesity in the general Japanese population is adjusted to BMI ≥ 25 kg/m^2^. Nevertheless, the appropriate reference values for the population with SCI have not been established. Previous studies have suggested BMI > 22 kg/m^2^ [43] as the SCI-specific cutoff values for identifying obesity. In 2014, Inayama et al. reported that the cutoff value for Japanese men with SCI for a VAT of 100 cm^2^ was a BMI of 22.5 kg/m^2^ [20]. However, regardless of the BMI criteria, there were discrepancies between the prevalence of obesity as identified by these BMI and %BF criteria for obesity. This finding is congruent with previous studies [4,7,44]. Routine whole-body DXA assessments may be necessary to accurately assess body composition and the associated cardiometabolic risks for individuals with SCI [7].

The present study has some limitations. First, this was a retrospective, cross-sectional study conducted at a single local hospital in Japan, which might limit the generalizability of our results. Second, there was no healthy control group as a reference to compare the prevalence of sarcopenia, obesity, and sarcopenic obesity; thus, only indirect comparisons were possible. Third, considering that no clear evidence exists regarding whether muscle atrophy following SCI could be assessed using the current definition of sarcopenia [11], our study identified sarcopenia using lean mass indices without muscle function assessment (strength or performance). To define sarcopenia in the elderly in the general population, it is recommended that muscle strength should be assessed by hand grip strength and that low physical performance should be evaluated by either a short physical performance battery, 6 m walk, or 5-time chair stand test [5]. However, these tests are not possible for persons with SCI, or the results might be biased [11]. For instance, the muscle strength of patients with tetraplegia could not be measured using dynamometers but this was possible in patients with paraplegia; nevertheless, the results might have been biased because wheelchair users are expected to have more upper limb strength than the general population as they depended on their arms for their ADLs. Physical performance tests might be possible in patients with incomplete injuries; conversely, these tests are not possible for the majority of patients with complete injuries. Alternative assessment tools for muscle function are thus required to accurately diagnose sarcopenia and sarcopenic obesity in the population with SCI.

## 5. Conclusions

The prevalence of sarcopenia, obesity, and sarcopenic obesity in our study sample were 76%, 85%, and 64%, respectively, suggesting that these conditions are prevalent in patients with SCI in Japan. Furthermore, various factors were found to be associated with lean and adipose indices. In particular, female sex, tetraplegia, motor-complete injury, and inability to walk were identified as risk factors for muscle atrophy comparable to sarcopenia. In addition, female sex was identified as a risk factor for obesity. Routine monitoring of body composition is necessary, especially in individuals with multiple risk factors, to identify those in need of preventive and therapeutic interventions.

## Figures and Tables

**Table 1:** Etiology of spinal cord injury.

		*n*	
Traumatic (*n* = 77)			
	Motor vehicle accidents	16	(21%)
	Falling from high heights	28	(36%)
	Falling on a level surface	20	(26%)
	Sports	1	(1%)
	Others	12	(16%)
Non-traumatic (*n* = 20)			
	Spinal cord infarction	11	(55%)
	Spinal epidural hematoma	4	(20%)
	Spinal epidural abscess	1	(5%)
	Transverse myelitis	4	(20%)
Values are presented as *n* (%).			

**Table 2:** Patients’ characteristics.

		Entire Patients *n* = 97		Tetraplegia *n* = 59		Paraplegia *n* = 38		*p*		Motor-Complete *n* = 31		Motor-Incomplete *n* = 66		*p*	
Sex, male		77	(79%)	46	(78%)	31	(82%)	0.668	c	25	(81%)	52	(79%)	0.833	c
Age (years)		64.0	(55–73)	66.0	(57.0–75.0)	62.5	(54.1–71.9)	0.532	b	60.2	±13.5	62.9	±11.7	0.315	a
Age at onset (years)		56.5	±15.3	62.0	(52.5–71.5)	56.0	(45.6–66.4)	0.353	b	52.0	(42.0–62.0)	62.5	(52.4–72.6)	0.005	b *
Duration after injury (years)		0.8	(0–5.26)	1.2	(0–6.0)	0.8	(0–4.5)	0.737	b	4.4	(0–11.9)	0.3	(0–3.0)	<0.001	b *
Etiology of injury															
	Traumatic	77	(79%)	56	(95%)	21	(55%)	<0.001	c *	24	(77%)	53	(80%)	0.743	c
	Non-traumatic	20	(21%)	3	(5%)	17	(45%)			7	(23%)	13	(20%)		
% Tetraplegia		59	(61%)	NA		NA				17	(55%)	42	(64%)	0.408	c
% AIS A/B		31	(32%)	17	(29%)	14	(37%)	0.408	c	NA		NA			
% Walker		50	(52%)	29	(49%)	21	(55%)	0.557	c	3	(10%)	47	(71%)	<0.001	c *
% Absence of dysphagia		92	(95%)	54	(92%)	38	(100%)	0.078	c	29	(94%)	63	(95%)	0.515	c
Anthropometrics															
	Height (cm)	165.4	±8.4	165.2	±8.4	165.6	±8.4	0.818	a	164.7	±7.5	165.7	±8.8	0.580	a
	Weight (kg)	62.3	(54.7–69.9)	59.0	(51.9–66.1)	66.8	(58.9–74.7)	0.002	b *	62.0	(56.4–67.7)	63.0	(53.4–72.5)	0.484	b
	BMI (kg/m²)	22.8	(20.1–25.6)	21.8	(19.4–24.2)	24.4	(22.4–26.4)	<0.001	b *	22.0	(19.0–24.9)	23.1	(20.5–25.7)	0.419	b
				**Females** ***n* = 20**		**Males** ***n* = 77**		***p***		**Non-walkers** ***n* = 47**		**Walkers** ***n* = 50**		***p***	
Sex, male				NA		NA				36	(77%)	41	(82%)	0.511	c
Age (years)				69.0	(60.9–77.1)	64.0	(54.8–73.3)	0.126	b	63.0	(53.0–73.0)	64.5	(56.3–72.8)	0.623	b
Age at onset (years)				63.5	(53.8–73.2)	57.0	(47.8–66.3)	0.116	b	54.0	(42.0–66.0)	61.5	(51.8–71.3)	0.059	b
Duration after injury (years)				1.0	(0–5.0)	0.8	(0–5.6)	0.786	b	3.9	(0–9.7)	0.3	(0–1.5)	0.002	b *
Etiology of injury															
	Traumatic			17	(85%)	60	(78%)	0.362	c	38	(81%)	39	(78%)	0.729	c
	Non-traumatic			3	(15%)	17	(22%)			9	(19%)	11	(22%)		
% Tetraplegia				13	(65%)	46	(60%)	0.668	c	30	(64%)	29	(58%)	0.557	c
% AIS A/B				6	(30%)	25	(32%)	0.833	c	28	(60%)	3	(6%)	<0.001	c *
% Walker				9	(45%)	41	(53%)	0.511	c	NA		NA			
% Absence of dysphagia				19	(95%)	73	(95%)	0.727	c	43	(91%)	49	(98%)	0.162	c
Anthropometrics															
	Height (cm)			154.5	±6.3	168.2	±6.2	<0.001	a *	165.1	±9.0	165.7	±7.8	0.721	a
	Weight (kg)			55.5	(47.5–63.5)	63.5	(55.6–71.4)	0.011	b *	59.0	(52.0–66.1)	65.3	(56.7–73.8)	0.044	b *
	BMI (kg/m²)			23.3	(19.7–26.8)	22.8	(20.4–25.3)	0.402	b	22.0	(18.6–25.4)	23.6	(21.5–25.8)	0.057	b

Values are presented as mean ± SD, median (IQR), or *n* (%). Abbreviations: AIS, American Spinal Injury Association Impairment Scale; BMI, body mass index; IQR, interquartile range; NA, not applicable; SD, standard deviation. a: *t*-test; b: Mann–Whitney *U* test; c: chi-squared test. * *p* < 0.05.

**Table 3:** DXA-measured body composition outcomes.

		Entire Patients *n* = 97		Tetraplegia *n* = 59		Paraplegia *n* = 38		*p*		Motor-Complete *n* = 31		Motor-Incomplete *n* = 66		*p*	
Lean mass indices															
	Whole-body lean mass (kg)	42.3	±8.5	40.1	±7.5	45.6	±9.1	0.002	a *	39.1	±7.1	43.8	±8.8	0.012	a *
	% Whole-body lean mass	66.5	±7.2	66.7	±7.5	66.3	±6.8	0.809	a	63.8	±6.7	67.8	±7.1	0.010	a *
	Trunk lean mass (kg)	22.5	±4.3	21.2	±3.6	24.5	±4.5	<0.001	a *	21.4	±3.6	23.0	±4.5	0.092	a
	Arm lean mass (kg)	4.7	(3.7–5.6)	4.3	±1.0	5.8	±1.5	<0.001	a *	4.9	±1.6	4.9	±1.4	0.917	a
	Leg lean mass (kg)	11.4	(8.95–13.8)	11.3	(8.8–13.7)	11.4	(8.8–14.0)	0.825	b	9.5	±2.4	12.5	±3.2	<0.001	a *
	ASM (kg/m²)	5.9	±1.2	5.9	(5.0–6.8)	5.8	(5.2–6.5)	0.049	b *	5.3	±1.0	6.3	±1.2	<0.001	a *
Adiposity indices															
	Whole-body fat mass (kg)	18.8	(14.8–22.9)	17.1	(13.6–20.7)	20.9	(17.1–24.7)	0.020	b *	19.2	(14.8–23.5)	18.4	(14.2–22.6)	0.225	b
	% Body fat	30.1	±7.6	29.9	±7.8	30.4	±7.3	0.758	a	32.9	±7.2	28.8	±7.5	0.012	a *
	Trunk fat mass (kg)	9.1	(6.8–11.5)	8.8	±3.4	10.8	±5.3	0.023	a *	9.1	(6.6–11.6)	9.1	(7.0–11.2)	0.178	b
	Arm fat mass (kg)	2.2	(1.6–2.7)	2.1	(1.6–2.6)	2.5	(1.9–3.1)	0.054	b	2.2	(1.6–2.8)	2.2	(1.7–2.7)	0.676	b
	Leg fat mass (kg)	5.9	(4.6–7.3)	5.9	±2.6	6.6	±2.3	0.172	a	6.5	(4.9–8.2)	5.7	(4.4–7.0)	0.171	b
	VAT (cm²)	123.4	(87.5–159.3)	111.5	(84.6–138.4)	142.3	(102.4–182.2)	0.034	b *	133.8	(95.0–172.6)	115.3	(79.9–150.6)	0.103	b
				**Females** ***n* = 20**		**Males** ***n* = 77**		***p***		**Non-Walkers** ***n* = 47**		**Walkers** ***n* = 50**		***p***	
Lean mass indices															
	Whole-body lean mass (kg)			33.2	±4.7	44.6	±7.7	<0.001	a *	39.4	±8.0	45.0	±8.2	<0.001	a *
	% Whole-body lean mass			57.6	±4.6	68.8	±5.8	<0.001	a *	64.4	±7.3	68.6	±6.6	0.003	a *
	Trunk lean mass (kg)			18.0	±2.5	23.6	±3.9	<0.001	a *	21.4	±4.2	23.5	±4.2	0.016	a *
	Arm lean mass (kg)			3.3	(2.6–4.1)	4.9	(4.1–5.7)	<0.001	b *	4.7	±1.5	5.0	±1.4	0.253	a
	Leg lean mass (kg)			8.6	±1.6	12.3	±3.1	<0.001	a *	9.5	(7.7–11.2)	12.7	(10.7–14.7)	<0.001	b *
	ASM (kg/m²)			5.1	±0.8	6.2	±1.2	<0.001	a *	5.2	(4.6–5.9)	6.5	(5.8–7.2)	<0.001	b *
Adiposity indices															
	Whole-body fat mass (kg)			22.7	(17.9–27.5)	18.0	(14.4–21.7)	0.003	b *	18.7	(13.2–24.2)	19.2	(15.6–22.7)	0.525	b
	% Body fat			39.5	±4.8	27.7	±6.2	<0.001	a *	32.3	±7.8	28.0	±6.9	0.005	a *
	Trunk fat mass (kg)			10.5	(8.0–12.9)	8.8	(6.7–11.0)	0.005	b *	8.9	(6.2–11.6)	9.3	(7.2–11.4)	0.453	b
	Arm fat mass (kg)			2.6	(1.6–3.6)	2.1	(1.7–2.6)	0.004	b *	2.2	(1.5–2.9)	2.2	(1.8–2.6)	0.665	b
	Leg fat mass (kg)			7.3	(5.7–8.8)	5.6	(4.3–6.9)	<0.001	b *	6.0	(4.2–7.8)	5.8	(4.6–7.0)	0.341	b
	VAT (cm²)			130.4	±54.0	122.9	±49.9	0.561	a	131.2	±55.7	118.1	±44.8	0.206	a

Values are presented as mean ± SD or as median (IQR). Abbreviations: BMI, body mass index; VAT, visceral adipose tissue; ASM, appendicular skeletal muscle mass; DXA, dual-energy X-ray absorptiometry; IQR, interquartile range; SD, standard deviation. a: *t*-test; b: Mann–Whitney *U* test. *p < 0.05.

**Table 4:** Prevalence of obesity, sarcopenia, and sarcopenic obesity based on the general and SCI-specific thresholds.

			Threshold	Entire (*n* = 97)		Male (*n* = 77)		Female (*n* = 20)	
Sarcopenia guidelines									
	AWGS recommendation								
		ASM	Male: <7.0 kg/m²	74	(76%)	60	(78%)	14	(70%)
			Female: <5.4 kg/m²						
Obesity guidelines									
	AACE recommendation								
		%BF	Male: >25%	66	(68%)	49	(64%)	17	(85%)
			Female: >35%						
	Japanese guidelines								
		VAT	≥100 cm²	62	(64%)	49	(64%)	13	(65%)
		BMI	≥25 kg/m²	28	(29%)	21	(27%)	7	(35%)
	SCI-specific threshold								
		%BF	Male: >22%	82	(85%)	65	(84%)	17	(85%)
			Female: >35%						
		BMI	>22.5 kg/m²	50	(52%)	40	(52%)	10	(50%)
			>22 kg/m²	56	(58%)	43	(56%)	13	(65%)
Sarcopenic obesity									
		ASM and %BF	Male: ASM < 7.0 kg/m², %BF > 25% Female: ASM < 5.4 kg/m², %BF > 35%	49	(51%)	38	(49%)	11	(55%)
			Male: ASM < 7.0 kg/m², %BF > 22% Female: ASM < 5.4 kg/m², %BF > 35%	62	(64%)	51	(66%)	11	(55%)
		ASM and BMI	Male: ASM < 7.0 kg/m², BMI ≥ 25 kg/m² Female: ASM < 5.4 kg/m², BMI ≥ 25 kg/m²	11	(11%)	8	(10%)	3	(15%)
		ASM and VAT	Male: ASM < 7.0 kg/m², VAT ≥ 100 cm² Female: ASM < 5.4 kg/m², VAT ≥ 100 cm²	43	(44%)	36	(47%)	7	(35%)

Values are presented as n (%). Abbreviations: AWGS, Asian Working Group for Sarcopenia; ASM, appendicular skeletal muscle mass; BMI, body mass index; VAT, visceral adipose tissue; %BF, percent body fat; AACE, American Association of Clinical Endocrinology; SCI, spinal cord injury.

**Table 5:** Multivariate linear regression analysis of DXA-measured sarcopenia and obesity-related outcomes.

		ASM				%BF				Whole-Body Lean Mass				Whole-Body Fat Mass			
		Standardized Coefficient				Standardized Coefficient				Standardized Coefficient				Standardized Coefficient			
Factors		β	*p*		VIF	β	*p*		VIF	β	*p*		VIF	β	*p*		VIF
	Sex (female/male)	0.34	<0.001	*	1.05	−0.63	<0.001	*	1.05	0.50	<0.001	*	1.05	−0.28	0.006	*	1.05
	Age	−0.11	0.211		1.12	0.03	0.746		1.12	−0.22	0.005	*	1.12	−0.08	0.425		1.12
	Duration of injury	−0.15	0.081		1.21	0.10	0.247		1.21	−0.08	0.303		1.21	0.02	0.868		1.21
	Lesion level (tetraplegia/paraplegia)	0.25	0.007	*	1.37	0.10	0.279		1.37	0.31	<0.001	*	1.37	0.28	0.018	*	1.37
	Severity (complete/incomplete)	0.20	0.043	*	1.58	−0.17	0.077		1.58	0.20	0.031	*	1.58	−0.01	0.959		1.58
	Ability to walk (non-walkers/ walkers)	0.29	0.006	*	1.63	−0.13	0.181		1.63	0.16	0.081		1.63	−0.11	0.372		1.63
	Dysphagia (absent/present)	0.03	0.716		1.13	−0.07	0.409		1.13	0.08	0.292		1.13	−0.04	0.726		1.13
	Etiology (traumatic/non-traumatic)	−0.02	0.865		1.40	−0.11	0.198		1.40	0.00	0.988		1.40	−0.13	0.275		1.40

Abbreviations: ASM, appendicular skeletal muscle mass; %BF, percent body fat; VAT, visceral adipose tissue; VIF, variance inflation factor. *p < 0.05.

## Data Availability

No new data were created or analyzed in this study. Data sharing is not applicable to this article.
